# Editorial

**Published:** 1904-07-15

**Authors:** 


### EDITORIAL.

The Four-Year Course.—The action of the National Association of Faculties at its meeting in Washington, June 9th, decided, by a vote of 24 to 21, schools present and voting, to continue the four-year course. The term was reduced to six months in place of seven. And the count system of allowing credits for entrance was adopted instead of so many terms or years of high-school training. In our judgment both of these latter were concessions if not actual retrograde steps. The count system is correct in principle, but so many alterative studies are admitted to make up the requisite counts, in number, that the new requirements do not rank with those formerly in use. A number of the schools represented are greatly displeased with the action in regard to the four-year course and threaten to resign membership in the Association and return to the three-year course. There seems to be one class of schools that contend that they can not teach longer than six months, because connected with medical schools which teach only six months, and when the medical students leave the dental students become dissatisfied and they have difficulty in keeping them at their work. Another class of schools in the Southern States think it is too warm down there to teach more than six months. And the Southern schools claim they have no such high-school system in the South as the Northern States, and they can't, therefore, advance the entrance requirements. These schools would rather teach four years of six months, and not exact higher entrance requirements, than to teach eight or nine months for three years with an advance of the entrance requirements to three years of high-school training or four years and graduation which is what a considerable number of the schools located in the northern part of the country want. Then there are a few who want a four-year course of nine months with a high-school graduation, or better, for admission. The problem is so complex that it is difficult of solution. The commercial aspect of the problem is also an important factor although it is not mentioned in the discussions. It is a fact that it will require a more expensive faculty and equipment to administer a four-year course than it will a three-year course, and many of the schools have about all they can do as it is to cover expenses. Some are, and have been, running at a considerable loss for years. There is no question in the minds of all but that some change in the system of training dentists must be adopted soon. Colleges must exact higher entrance qualifications, or materially improve the methods and quality of instruction. The four-year course may do it, but if this is overthrown something else must be substituted.

---

THE FACULTIES ASSOCIATION AGAIN CALLED TO READJUST THE FOUR-YEAR COURSE.—The Executive Committee has called the National Association of Dental Faculties to meet again July 18th, at St. Louis, to reconsider the action taken at Washington to continue the four-year course. The call states that so many of the schools are resigning from the Association that it is likely to be disorganized, and the cause of dental education get a great backset. No meeting can legally be held at this time, but it is thought that the importance of the matter justifies extraordinary measures. At this writing it seems entirely probable that the Association will decide to return to a three-year course. In fact, a considerable number of schools have already publicly made this announcement, and resigned their memberships in the Association. It looks as though there was going to be done a piece of work that we shall all be very sorry for in the future. The best interests of the cause of education ought to have more weight than personal interest.

Dr. William H. Whitslar has resigned his position as Professor of Pathology and Secretary of the Dental Department of Western Reserve University. This will be a distinct loss to the profession as well as the school, as Dr. Whitslar was a close student and a faithful instructor. He held the highest educational ideals, and it seems that he should, by all means, remain a teacher. This department of instruction has been sadly depleted during the past year by the death of Drs. Taft, Barrett, and Flagg, and it does seem as though the men who are capable and actively at work in this department should be kept at it. We know however that such a man as Dr. Whitslar loves his profession too much to take this step without good reasons, and that he will not therefore stop contributing his work to the profession through other channels.

